# Alpha-Mannosidosis: Therapeutic Strategies

**DOI:** 10.3390/ijms19051500

**Published:** 2018-05-17

**Authors:** Maria Rachele Ceccarini, Michela Codini, Carmela Conte, Federica Patria, Samuela Cataldi, Matteo Bertelli, Elisabetta Albi, Tommaso Beccari

**Affiliations:** 1 Department of Pharmaceutical Sciences; University of Perugia, Via Fabretti 48, 06123 Perugia, Italy; chele@hotmail.it (M.R.C.); michela.codini@unipg.it (M.C.); carmela.conte@unipg.it (C.C.); patriafederica@gmail.com (F.P.); samuelacataldi@libero.it (S.C.); elisabetta.albi@unipg.it (E.A.); 2 MAGI Human Medical Genetics Institute; laboratory of genetic diagnosis of rare diseases, 38068 Rovereto, Italy; matteo.bertelli@assomagi.org

**Keywords:** alpha-d-mannosidase, alpha-mannosidosis, enzyme replacement therapy, lysosomes

## Abstract

Alpha-mannosidosis (α-mannosidosis) is a rare lysosomal storage disorder with an autosomal recessive inheritance caused by mutations in the gene encoding for the lysosomal α-d-mannosidase. So far, 155 variants from 191 patients have been identified and in part characterized at the biochemical level. Similarly to other lysosomal storage diseases, there is no relationship between genotype and phenotype in alpha-mannosidosis. Enzyme replacement therapy is at the moment the most effective therapy for lysosomal storage disease, including alpha-mannosidosis. In this review, the genetic of alpha-mannosidosis has been described together with the results so far obtained by two different therapeutic strategies: bone marrow transplantation and enzyme replacement therapy. The primary indication to offer hematopoietic stem cell transplantation in patients affected by alpha-mannosidosis is preservation of neurocognitive function and prevention of early death. The results obtained from a Phase I–II study and a Phase III study provide evidence of the positive clinical effect of the recombinant enzyme on patients with alpha-mannosidosis.

## 1. Introduction

Glycoproteins are widely present within cells and cell surfaces. Two pathways are used to synthetize glycoproteins producing O-linked oligosaccharides or N-linked oligosaccharides. The oligosaccharides are degraded in the lysosomes by a series of exoglycosidases acting from the non-reducing end [1]. The low pH of the lysosomes not only provides the optimal conditions for the enzyme activities but will probably partially denature the glycoproteins. The enzyme aspartylglycosaminidase is responsible for the hydrolysis of *N*-acetylglucosamine–asparagine linkages, and the enzyme di-*N*-acetylchitobiase hydrolyzes the *N*-acetylglucosamine residues from the reducing end [2]. Subsequently, the glycoprotein carbohydrate chain is degraded by sequential removal of monosaccharides. Each step in this catabolic pathway depends on a specific enzyme including the removal of posttranslational modifications such as sulfation, phosphorilation, or esterification. Very little is known about the precise catabolism of the polypeptide portion of any glycoprotein in vivo. Deficiency of an enzyme involved in the catabolic pathway of N-linked glycans leads to the accumulation of the respective substrate and consequently to the onset of a specific storage disorder [3]. Di-*N*-acetylchitobiase and core specific α1–6 mannosidase represent the only exception and so far no diseases have been associated to the lack of these enzymes. For many of these disorders, the accumulation of the undigested material results in vacuolization in cells such as peripheral blood cells and fibroblasts. This accumulation can be also responsible for pleiotropic effects on cellular functions such as synaptic release, exocytose, and autophagy. Among glycoproteinoses, alpha-mannosidosis is caused by the deficiency of the lysosomal enzyme alpha-d-mannosidase (EC 3.2.1.24). Lysosomal α-mannosidase activity (EC 3.2.1.24) with an acidic pH optimum is ubiquitous in human tissues where it occurs as two major forms, A and B, that can be separated by ion-exchange chromatography on Diethylaminoethyl-cellulose (DEAE-cellulose) [4]. The A and B forms are the product of a single gene (*MAN2B1*), as demonstrated in the lysosomal storage disease alpha-mannosidosis (MIM 248500) in which both A and B are lacking [5]. This enzyme has been characterized and purified from several mammalian sources including humans [6]. Lysosomal alpha-d-mannosidase from humans, rats, cattle, and cats can catalyze the hydrolysis of alpha(1,2)-, alpha(1–3)-, and alpha(1–6)-mannosidic linkages present in the N-linked oligosaccharides. Alpha-mannosidosis as a consequence of a decreased activity of alpha-d-mannosidase was first described in 1967 resembling Hurler syndrome (MIM number 607014), but the storage material was not acid mucopolysaccharide [7]. Alpha-mannosidosis has been described also in cattle (*Bos taurus*) [8], cats (*Felis catus*) [9], and guinea pigs (*Cavia porcellus*) [10]. In addition to the natural occurring alpha-mannosidosis, the disease was induced experimentally with swainsonine in the sheep (*Ovis aries*) [11] and a mouse model of alpha-mannosidosis was generated by homologous recombination [12]. The histological and biochemical characteristics underline the similarity between alpha-mannosidosis in human and mice, whereas the clinical presentations are different. The phenotype in the alpha-mannosidase deficient mice appear attenuated compared to the human phenotype. Anyway in knockout (KO) mice, there is a drastically increased urinary excretion of mannose-containing oligosaccharides, as observed in human alpha-mannosidosis. Neutral sugars are increased and oligosaccharides are accumulated in several tissues such as spleen, kidney, brain, and liver. This mouse alpha-mannosidosis model has been more useful than other models such as cat and cattle in terms of life span, ease of breeding and control of genetic background. The mouse model has been used to investigate the pathogenesis of the disease and to develop an enzyme replacement therapy (ERT) for alpha-mannosidosis.

## 2. Inherited Deficiency of Alpha-Mannosidase (Alpha-Mannosidosis)

Alpha-mannosidosis is a rare lysosomal storage disorder with an autosomal recessive inheritance. Alpha-mannosidosis shows a birth incidence of about 1:1,000,000 in the Netherlands, Portugal and Australia, of 1:1,042,804 in the Czech Republic [13] and 1:600,000 in Norway [14]. The children are often born apparently normal, and their condition worsens progressively. Alpha-mannosidosis is a progressive disorder showing as clinical features mental retardation, hearing loss, skeletal deformities, central nervous system involvement, and immuno defects. Traditionally, alpha-mannosidosis has been classified in two groups: Type 1, with a mild phenotype including hearing loss, mental retardation, and survival into adulthood, and Type 2, with a severe phenotype showing hepatosplenomegaly and early death following severe infections [15]. Recently, based on published cases, three clinical types have been suggested. Type 1 is a mild form, clinically recognized after 10 years of age, without skeletal abnormalities and very slow progression; Type 2 is a moderate form, clinically recognized before 10 years of age, with skeletal abnormalities and slow progression with developmental of ataxia at age 20–30; Type 3 is a severe form, immediately recognized, with skeletal abnormalities and obvious progression, leading to an early death from primary central nervous system involvement or myopathy [14]. Most patients belong to Type 2. Diagnosis is made by measuring acid alpha-mannosidase activity in leukocytes or other nucleated cells using artificial colorimetric of fluorimetric substrates. In affected individuals, lysosomal alpha-mannosidase activity in leukocytes is 5–15% of normal activity. Residual activity may be due to other non-lysosomal alpha-mannosidase expressed in the cells such as Golgi alpha-mannosidase and cytosolic alpha-mannosidase. If the lysosomal alpha-mannosidase is immunoprecipitated, enzyme activity in affected patients ranges from 0.1 to 1.3% [16]. The lysosomal alpha-mannosidase activity in carrier is usually 40–60% of normal activity and therefore cannot be used for carrier detection. The diagnosis is then confirmed at the molecular level by sequencing both strands all 24 exons. Elevated urinary secretion of mannose-rich oligosaccharides determined by thin-layer chromatography or high-performance liquid chromatography (HPLC) is suggestive but not diagnostic. In order to characterize the clinical features and disease progression of patients affected by alpha-mannosidosis, a longitudinal study was conducted on 43 patients from four different European countries [15]. The age range of the patients was 3–42 years. For each participant, a medical history, a complete physical and neurological examination, and a joint range of motion and assessment of physical endurance and of lung function were completed. In addition, serum and urinary oligosaccharide levels were determined. The longitudinal study was also conducted in order to assess the natural history of patients with alpha-mannosidosis and to evaluate short-term (24 months) changes in the disease parameters. Few retrospective studies on the clinical course of alpha-mannosidosis had been performed, and all of them included only a small number of patients [17,18,19,20,21]. A feature found in the majority of patients in this study is abnormality of the musculoskeletal system. The severity of bone deformities did not change over the time. Ataxia and mental retardation were the most noticeable findings observed in the study. All patients with alpha-mannosidosis, independently from age, showed hearing loss (bone and conductive). Minor ophthalmological abnormalities were observed such as slight corneal opacities or cataract. In the study, lung function tests were performed in the majority of the patients. In the younger age group, there was a decline in the percentage of predicted forced vital capacity (FVC) within the observation time of 2 years. The older patients, compared with the children, showed a lower percentage of predicted FVC at baseline. Another method used in this study was the 6 min walking test (6-MWT), which allows for the measure of the general endurance of the patients without obtaining clear results. The same conclusion has been obtained with the 3 min stair climb test. Patients affected with alpha-mannosidosis excrete high amounts of undegraded oligosaccharides in the urine and plasma that interestingly correlate with the 6-MWT. In fact, patients with an elevated oligosaccharides excretion showed an impaired ability to walk. A similar correlation was found with the stair climb test. It can be concluded that the severity of the disease might depend on the level of the secreted oligosaccharides. Therefore, urinary oligosaccharides can be used as a surrogate marker in clinical trials such as for enzyme replacement therapy. A similar correlation was found in patients affected by mucopolisaccharidoesis type VI [22].

## 3. Genetics of Alpha-Mannosidase

The gene encoding human alpha-d-mannosidase (MAN2B1b) has been localized close to the centromere 19 (19p133,2–q129) by analysis of human/rodent cell hybrids for alpha-mannosidase activity [23,24] and by PCR screening of a human/rodent mapping cell hybrid mapping panel DNA [25,26]. The exact genomic localization on chromosome 19 of the *MAN2B1* gene has been determined using the human genome sequence (chr19:12,646,508–12,666,777). A partial sequence of a cDNA encoding for the human alpha-mannosidase was reported in 1994 [25], and two full-length cDNAs were later isolated and sequenced [26,27] and the first mutation identified [27]. The deduced amino acid sequence contains two potential translation initiation signals. The nucleotide sequence surrounding both ATG is very similar to the Kozak consensus sequence for translation initiation [28]. The exon–intron structure of the human gene has been determined [29,30]. The gene is 21.5 kb long and is divided in 24 exons. In silico analysis of the 5′ flanking region of the gene shows a high GC content and has two Sp1 and three AP-2 sites typical of housekeeping genes. The transcription start site, determined by 5′ RACE appears to be in positions -28 and -20 from the first ATG. cDNAs encoding for the mouse [6], bovine [31], cats [32], guinea pigs [33], and lysosomal alpha-d-mannosidase have been isolated and sequenced. The MAN2B1 cloning and its structure have allowed for the identification and characterization of many mutations causing alpha-mannosidosis [34,35,36,37,38,39,40,41,42,43]. Very recently, all genetic variants in the MAN2B1 gene were reported in the database Amamutdb.no [44]. The database offers structural and relational information on MAN2B1 mutations and genotypes along with the associated clinical phenotypes. In the database, 155 variants from 191 patients are present. All these data have greatly improved the molecular diagnosis of alpha-mannosidosis, but there is still no clear relationship between genotype and phenotype. The lack of a relationship between genotype and phenotype is a common feature in the lysosomal storage disorders.

## 4. Therapy of Alpha-Mannosidosis

### Bone Marrow Transplantation

Hematopoietic stem cell transplantation (HCT) is an effective therapy for selected inherited metabolic diseases with associated neuronal dysfunction, such as Hurler syndrome and cerebral X-linked adrenoleukodystrophy [45]. In bone marrow transplantation, normal donor stem cells differentiate to different lineages that colonize many organs and tissues. Secretion of the normal enzyme by these cells followed by uptake by other cells of perhaps direct transfer of the normal enzyme mediated by cell-to-cell contact results in wide distribution of the therapeutic enzyme. HCT at the moment is the only clinically available therapeutic approach to enzyme replacement. HCT has been tested for the first time in cats with alpha-mannosidosis [46]. This study indicated that HCT can lead to significant levels of alpha-mannosidase within neurons of the central nervous system and to the compensation for the genetic metabolic defect. HCT for alpha-mannosidosis was first reported in two patients: one patient died 18 weeks after transplantation [47], whereas in the other patient the therapy led to a complete regression of organomegaly with improvement in bony disease but it was too early to see if it was preventing the neurological deterioration [48]. This the first clinical case of mannosidosis in which clinically positive effects were obtained. Two additional studies on HCT have been reported: one on four patients with alpha-mannosidosis [49] and the second one was the first patient who received a T-cell-depleted peripheral blood stem cell transplantation (PBSCT) for alpha-mannosidosis [50]. All four patients received unrelated donor graft. All four patients showed normalization of leukocyte alpha-mannosidase activity after HCT. Intellectual function was stabilized, with improvement in adaptive skills and verbal memory function in three of four patients. No new skeletal abnormalities were observed. Hearing was improved to normal or near normal for speech frequencies in three patients. The patient that received PBSCT showed not only a stabilized neurological status but also good progress in his motor, social, and intellectual capabilities as well as in his speech development. The mother was chosen as stem cell donor despite the fact that she was a heterozygous carrier of alpha-mannosidosis with enzyme activity levels below normal. Therefore, heterozygous carriers (i.e., parents and siblings) can be considered as suitable donors. The effects of early and late bone marrow transplantation has been tested in two brothers with alpha-mannosidosis [51]. The older brother underwent transplantation at 13 years for the treatment of increasing somatic problems and recurrent infections. The younger brother had undergone transplantation pre-symptomatically at 6 months of age. In the oldest boy, transplantation led to a resolution of diarrhea and a decrease in infections. However, the most noticeable result was his improvement in concentration and communication as observed in the post-transplantation course in the oldest of the patients reported by Grewal et al. [48]. The youngest boy showed very few complications after HCT and has progressed well, attending mainstream school. Anyway, his hearing problems were not resolved. No neurological progression of the disease was observed and his overall progress was excellent. More recently, a successful unrelated bone marrow transplantation in two siblings with alpha-mannosidosis has been reported [52]. In both siblings, enzyme levels reached normal limits, and improvements in clinical symptoms were recognized early after HCT. The largest retrospective analysis of patients with alpha-mannosidosis after HCT has been reported by Mynarek [53]. It has been shown that HCT in patients with alpha-mannosidosis is a feasible therapeutic option. The mortality and morbidity after HCT was comparable to other diseases. No clear mannosidosis-specific adverse events in the procedure were observed. Developmental improvement after HSCT was observed in all patients. The capacity to live an independent life has been reported in one patient. Improvement in hearing ability was seen in some patients without a total resolution of hearing disability. The most important clinical problem for alpha-mannosidosis patients was their deficiency in hearing and expressive speech. Stabilization or even improvement of skeletal abnormalities was reported by some treating physicians despite the difficulty of quantifying skeletal abnormalities in the growing skeleton. The primary indication to offer HCT in patients affected by alpha-mannosidosis is the preservation of neurocognitive function and the prevention of early death. The morbidity and mortality rate associated with HCT must be balanced. In the case of alpha-mannosidosis specific values for transplantation-related mortality rates are not available. Patients who have received transplants for metabolic diseases have also been found to be at higher risk of autoimmune haemolytic anemia [54] and pulmonary complication [55]. The procedure will become safer with increasing experience of HCT, as already seen in Hurler’s disease [56]. In conclusion, HCT should be considered a therapeutic approach in patients with alpha-mannosidosis.

## 5. Enzyme Replacement Therapy

In January 2018, the European Medicines Agency’s (EMA) committee for medical products for human USE (CHMP) recommended granting a marketing authorization in the European Union (EU) for Lamzede (Velmanase alfa) for long-term ERT in adults, adolescents, and children with mild–moderate forms of alpha-mannosidase. In EMA authorization, it is clearly stated that this recombinant enzyme does not cross the blood–brain barrier (BBB), as all other recombinant lysosomal enzymes so far approved for ERT. ERT is the most widely used therapy for lysosomal storage disorders, and it is now available worldwide for about 15 different diseases [57]. However, there are still obstacles to successful ERT, such as immune reactions against the infused enzyme, the mistargeting of enzymes rather than lysosomes, and intractable tissues. The beginning of the ERT for alpha-mannosidosis dates back to 2004, when its efficacy was tested in alpha-mannosidosis mice [58] that were generated by homologous recombination [12]. The generation of a mouse model for alpha-mannosidosis and the production of the recombinant alpha-mannosidase have made it possible to study the efficacy of ERT in alpha-mannosidosis. Homozygous mutant mice show alpha-mannosidase deficiency and elevated urinary secretion of mannose containing oligosaccharides. Thin-layer chromatography revealed an accumulation of oligosaccharides in liver, kidney, spleen, testis, and brain. The cellular alterations were characterized by multiple membrane-limited cytoplasmic vacuoles as seen for instance in the liver, exocrine pancreas, kidney, thyroid gland, smooth muscle cells, osteocytes, and neurons of the central and peripheral nervous system. The morphological lesions and their topographical distribution, as well as the biochemical alterations, closely resemble those reported for human alpha-mannosidosis. Therefore, the mouse model for alpha-mannosidosis is a suitable tool to study the phatophysiology of the disease and to develop therapeutic strategies. Three different enzyme were tested after intravenous administration: the lysosomal acid alpha-mannosidase from bovine kidney, and human and mouse recombinant alpha-mannosidase. The bovine and human enzymes were barely phosphorylated, whereas the bulk of the mouse LAMAN contained mannose 6-phosphate recognition markers. The clearance decreased from bovine to human to mouse alpha-mannosidase with half-times of 4, 8, and 12 min, respectively. The apparent half-life of the internalized enzyme was dependent on the enzyme source as well as tissue type and varied between 3 and 16 h. The human enzyme showed the best half-life. The corrective effect on the storage of neutral oligosaccharides was time-, tissue-, and dose-dependent, and the effects were observed to be transient. The neutral oligosaccharides started to reaccumulate 2–6 days after a single injection. The human enzyme proved to be the most effective. A remarkable correction of storage oligosaccharides was observed in the liver, kidney, and heart. The corrective effect was most pronounced in the liver, the tissue showing the highest alpha-mannosidase activity when compared with wild-type mice. More importantly, the concentration of neutral oligosaccharides also decreased in the brain by about one-fourth. Furthermore, in the brain homogenates of treated mice, some alpha-mannosidase activity was observed. In the alpha-mannosidosis mice, the integrity of the BBB was preserved. Intravenously administrated Evans blue did not cross the BBB, and immunoglobulin G was excluded from extra vascular tissue. For this reason, the hypothesis was that the decreased oligosaccharide storage in the brain was due to the clearance of oligosaccharides via the blood circulation or via an improved flow of the cerebrospinal fluid. This promising effect on the brain tissue was investigated a few years later in an effort to understand the mechanism of recombinant alpha-mannosidase entry in cells, especially those of the central nervous system (CNS) [59]. The study was performed using low phosphorylated human recombinant alpha-mannosidase (rhLAMAN) in alpha-mannosidosis mice. Low phosphorylation content was shown to correlate with an increased half-life of recombinant enzymes such as beta-glucuronidase [60] and alpha-mannosidase [61]. Low doses of rhLAMAN (25 U/kg) were able to significantly reduce storage in peripheral tissues. The storage reduction was also obtained in neurons of the CNS using a high dose of rhLAMAN (500 U/kg). After this treatment, low levels of rhLAMAN activity (14.8% of the wild type level) were determined in the brains of injected KO mice. Using repeated injections of rhLAMAN (500 U/kg), oligosaccharides were reduced by more than 50% in the brain. To demonstrate that this reduction was a consequence of the uptake of rhLAMAN into the cells, mice were injected with a very high dose (1000 U/kg). The presence of the recombinant enzyme inside the cell was demonstrated by an immunohistological approach using a specific antibody. All together these results indicate that the enzyme not only crossed the BBB but was also taken up in the neurons. However, immunological response to the recombinant enzyme was associated with high mortality precluding long-term ERT in alpha-mannosidosis mice. Other studies suggest that lysosomal enzymes may cross the BBB in animal models with Krabbe disease [62], metachromatic leucodystrophy [63], mucopolisaccharidosis type I [64], and mucopolisaccharidosis type VII [65]. An interesting hypothesis is that the recombinant enzymes may cross the BBB and reach the brain by endocytosis when used at high doses [64]. High doses allow the enzyme to be available for longer period in the serum and therefore to be taken up by pinocytosis that it is not very efficacious in the BBB. High doses are probably necessary because the injected enzyme is quickly captured by visceral organs and thus the bioavailability of the enzyme is rapidly reduced. Recently, to overcome the immunological problems associated with ERT in alpha-mannosidosis mice, an immune-tolerant mouse model of alpha-mannosidosis was generated [66]. This mouse model allowed for an evaluation of the effect of high-dose long-term ERT on CSF pathology using rhLAMAN. The immune-tolerant mice show biochemical and behavioral characteristics similar to conventional KO mice. The long-term ERT resulted in a decrement of the oligosaccharides accumulation in the brain, normalization of alpha-mannosidase activity and morphology, and a decrease in microglial activation. In addition, an improvement of cognitive deficits and exploratory activity were observed. There was an uptake of rhLAMAN in different types of nervous systems. This uptake is primarily M6P-independent. Biochemical evidence obtained from the ERT in KO mice suggested that hippocampus may be one of the brain structures that can benefit from long-term ERT. To this end, ERT was initiated in 2-month-old immune-tolerant alpha-mannosidosis mice and continued for 9 months. During the course of treatment, mice were trained in the Morris water maze task to assess spatial-cognitive performance, which was related to synaptic plasticity recordings and hippocampal histopathology. Long-term ERT reduced primary substrate storage and neuroinflammation in the hippocampus and improved spatial learning after mid-term (10 weeks+) and long-term (30 weeks+) treatment. Long-term treatment substantially improved the spatial-cognitive abilities of alpha-mannosidosis mice, whereas the effects of mid-term treatment were more modest. Detailed analyses of spatial memory and spatial-cognitive performance indicated that even prolonged ERT did not restore higher cognitive abilities to the level of healthy mice [67].

Recently, the results of a Phase I–II study to evaluate safety and efficacy of rhLAMAN (Zymenex 2012) after 12 months of treatment have been published [68]. Ten patients, age 7 to 17 years, were treated. In Phase I, the patients, divided into five different groups, received different intravenous doses. In Phase II, the patients were randomly divided into two groups, receiving either 25 or 50 U/kg of rhLAMAN. No statistically significant or clinically relevant differences were found between the two dosing groups. A statistically significant improvement was found in the 3 min stair climbing test (3-MSCT) at a mean of 30 steps but not in the 6-MWT. CSF-MAN2 oligosaccharides and biomarkers decreased during the 12 months of ERT. RhLAMAN was usually well tolerated. Two patients developed rhLAMAN antibodies and had moderate infusion-related reactions. No improvement in the hearing sensitivity related to ERT was observed in the treated patients. In this study, the efficacy of ERT was not correlated with the genotype of the patients or to their residual enzyme activity. The overall data of this study indicates that ERT with rhLAMAN is safe and may be used for the therapy of alpha-mannosidosis. The latest result on the ERT for alpha-mannosidosis comes from a Phase III study [69]. In this study, 14 adult patients [mean age 24.6 ± 5.3 years at baseline] participated in the Phase III study and long-term follow-up. The last observation was at 18 or 36 months of exposure.

The results show a statistically significant reduction of serum oligosaccharides from the baseline of 57.6% ± 30.5% at last observation.

Further, results of the 3-MSCT suggest a stabilization of the disease (baseline 53.0 ± 11.8 steps/min, last observation 53.6 ± 14.6 steps/min).

The study also noted that three out of five patients that required assistance walking at baseline did not need assistance following treatment.

Clinically relevant improvements were also observed in the Childhood Health Assessment Questionnaire Pain Index.

The authors concluded that initiating treatment with rhLAMAN in adulthood has proven to be effective in improving key disease biomarkers and in slowing the disease progression in multiple domains. Long-term efficacy of rhLAMAN was also investigated in 33 patients (19 pediatrics, 14 adults) treated for up to 48 months in Phase I–III studies and long-term follow-up [70]. The Bruininks–Oseretsky test (BOT-2) was a secondary efficacy endpoint used to capture fine and gross motor proficiency.

Across all treated patients, the mean BOT-2 total point score improved at Month 12 by +7.5 (*p* < 0.05) and at last assessment by +5.1 (*p* = 0.230) compared to baseline scores. Analysis by age showed a trend for greater change in the pediatric than adult patients. Pediatric patients appeared to show some improvements in fine motor precision, manual dexterity, upper limb co-ordination, and bilateral co-ordination. The authors concluded that, overall, these data provide evidence of the positive clinical effect of rhLAMAN. However, there is no clinical proof of the passage of rhLAMAN through the BBB, and the recombinant enzyme is not indicated for neurological involvement, since its efficacy in cognitive function was not proved. At present, there are no studies addressing the possibility of using pharmacological chaperons for the therapy of alpha-mannosidosis. Pharmacological chaperons therapy (PCT) might become an alternative to ERT for alpha-mannosidosis in the near future, as has already observed for Fabry disease [71]. Pharmacological chaperons selectively bind to misfolded enzymes in the endoplasmic reticulum, facilitating the correct folding of the protein and inducing functional recovery. Small molecules can cross the BBB; so PCT has emerged as promising for lysosomal diseases affecting the CNS.
